# Women report higher pain intensity at a lower level of inflammation after knee surgery compared with men

**DOI:** 10.1097/PR9.0000000000000595

**Published:** 2017-04-20

**Authors:** Nina Solheim, Simon Östlund, Torsten Gordh, Leiv Arne Rosseland

**Affiliations:** a Department of Anesthesia, Lovisenberg Diakonal Hospital, Oslo, Norway; b Department of Surgical Sciences, Pain Medicine, University of Uppsala, Uppsala, Sweden; c Department of Surgical Sciences, Pain Medicine, Uppsala University Hospital, Uppsala, Sweden; d Department of Research and Development, Division of Emergencies and Critical Care, Oslo University Hospital, Oslo, Norway; e Faculty of Medicine, Institute of Clinical Medicine, University of Oslo, Oslo, Norway

**Keywords:** Pain, Knee surgery, Inflammation, MMP-10, IL-8, Sex

## Abstract

**Introduction and Objectives:**

We previously found that women report more pain after knee arthroscopic procedures than men. It remains unclear whether this is due to different biochemical responses or nociceptive mechanisms.

**Methods:**

We analyzed acute pain-related inflammatory markers in a clinical model of patient self-reported pain immediately after knee surgery. To simultaneously measure 92 inflammatory biomarkers, we used the proximity extension assay with the Proseek Multiplex Inflammation I panel (Olink Bioscience, Uppsala, Sweden). Knee surgery was performed under general anesthesia with propofol and remifentanil. Analgesic drugs were only administered on patient request.

**Results:**

Women were 4.9 times more likely to report moderate or severe pain than men (95% confidence interval, 1.2–19.6, *P* = 0.024). Patient age, preoperative pain, and surgery duration were not significant factors. We analyzed synovial fluids from 44 patients (23 women, 21 men). After false discovery rate correction, MMP-10 was the only biomarker that was higher among men (*P* = 0.01). Linear discriminant analysis showed that 3 proteins (IL-8, CCL-4, and MCP-2) were expressed at higher levels in men, with differences of >1 normalized protein expression. No proteins were overexpressed by >1 normalized protein expression in women.

**Conclusion:**

Acute pain after knee arthroscopy was more intense in women, but pro-inflammatory biomarkers and MMP-10 were higher in men. Further knowledge of cytokine function is required before concluding that the disparities in biomarker expression are clinically unimportant. The similar biochemical signaling between sexes suggests that central mechanisms are of greater importance in sex-specific joint pain perception.

## Abstract 2

Women report more pain than men after arthroscopic surgery, but analyses of pro-inflammatory cytokines indicates higher male concentrations of pain biomarkers in synovial fluid.

## 1. Introduction

Acute postsurgical pain intensity varies substantially among patients^8^ and correlates with concentrations of inflammatory biomarkers, such as prostaglandin E_2_, proinflammatory cytokines, and chemokines.^34^ Prostaglandins, cytokines, chemokines, and neurotransmitters are clearly involved in the intricate communication between the immune system and the adaptable nervous system, and some reportedly play important roles in pain sensation mechanisms.^18^ Observational studies reveal a significant discrepancy between men and women regarding perceived acute pain following standardized surgical procedures.^7,26^ However, it is not clear whether this sex difference is due to dissimilar expressions of biochemical mediators.

Research findings over the past 2 decades consistently show a significant discrepancy between male and female pain responses, promoting increased enthusiasm for investigating the relationships between sex, sex, and pain.^7^ Compared with men, women seem to be at a higher risk of developing pain—commonly experiencing more intense acute pain and pain of longer duration, and more frequently reporting painful conditions at diverse body sites.^3^ Female sex is a predictor of acute pain and of chronic conditions, such as fibromyalgia, migraine, tension-type headaches, and irritable bowel syndrome.^29^ The reason for the difference between the sexes in experienced acute postoperative pain remains unknown. One suggested explanation is that men and women differ in their susceptibility to the opioids given during and after surgery, with women generally experiencing lower analgesic effect and thus greater pain.^35^ However, contradicting this theory, recent studies demonstrate a persisting sex gap after perioperative administration of short-working anesthetics without postoperative treatment and, thus, without clinically significant carryover effects.^5^

The very early immune response to a noxious stimulus, especially a nerve lesion, involves neutrophil granulocytes, which can be attracted by nerve growth factor, chemokine ligands 1 and 2 (CXCL1 and CXCL2), interleukin-8 (IL-8 and CXCL8), and leukotriene-B4.^37^ Macrophages enhance proinflammatory signaling through the expression of many pain mediators, including reactive oxygen species and cyclooxygenase-derived prostaglandins, which directly sensitize primary afferents.^22^ In cases of neuronal damage, proinflammatory signaling is further enhanced by glial cells that secrete potential hyperalgesia mediators, including tumor necrosis factor–alpha (TNF-α) and interleukins 1 and 6.^33^ The interactions between sensory neurons, immune cells, and glial cells are largely intertwined, and are mediated by proinflammatory cytokines, eg, interleukin 1β, 6, 12, and 18; interferon-γ (IFNγ); and TNF-α. These interactions are also influenced by the so-called natural anti-inflammatory cytokines, including IL-10, IL-4, IL-1 receptor antagonist (IL-1ra), and transforming growth factor–β (TGF-β).^30^ In addition, synovial fluid matrix metalloproteinase (MMP) activity is reportedly increased after meniscal tear compared with controls.^15^

We have previously shown that women report more pain after arthroscopic knee procedures than men.^26^ For decades, researchers have wondered whether this difference results from different biochemical responses or nociceptive mechanisms, and the pathophysiological circumstances have not yet been explained. In this study, we aimed to analyze a wide range of inflammatory markers related to acute pain in a clinical model of patient self-reported pain immediately after knee surgery.

## 2. Methods

The study protocol and biobank were approved by the Medical Ethics Committee for Health Region South East in Norway (2009/1261a). The protocol conformed to the Declaration of Helsinki, and the study was conducted following good clinical practices. All patients gave their consent to participate in this observational substudy and in a randomized clinical trial that is registered in an international registry of interventional studies (clinicaltrials.gov identifier: NCT00774540).

### 2.1. Patients

**Figure 1.**
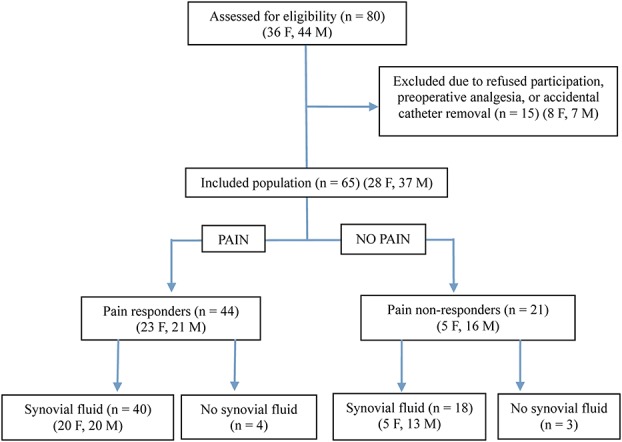
Flow chart of the numbers of patients screened, included, excluded, and analyzed. F, female; M, male.

For this study, we recruited 80 patients who were scheduled for day-case knee arthroscopic procedures at Lovisenberg Diakonale Hospital. Patients were invited to participate if they were older than 18 years, of American Society of Anesthesiologists (ASA) physical status classes 1 and 2, and could understand the information provided in Norwegian. Exclusion criteria were known intolerance or contraindications for ketorolac; alcohol or drug abuse; pregnancy or breastfeeding; comprehensive arthrosis or synovitis; renal failure (s-creatinine >160); heart failure with easily provoked symptoms; active gastric/duodenal ulcer; bleeding or perforation; increased bleeding tendency; liver cirrhosis; simultaneous participation in other trials; preoperative treatment with glucocorticoids, paracetamol (within 12 hours), nonsteroidal anti-inflammatory drugs (within 12–24 hours), or COX2 inhibitors (within 24–48 hours); intraoperative tourniquet use; preoperative pain of moderate to severe intensity; or risk for drug interactions with ketorolac. In addition, patients were recommended to withdraw from the study if they experienced very severe pain immediately after surgery. The final observational study sample included 65 subjects (28 women, 37 men) (Fig. 1). Synovial fluid samples were collected when the patients experienced moderate or severe pain on a 5-item verbal rating scale with the alternatives no, mild, moderate, severe, and very severe pain until 120 minutes after the end of surgery. Patients who experienced no or mild were observed 120 minutes before discharge.

### 2.2. Pain assessment

Preoperatively, the patients were given instructions for assessing pain intensity on a 0–100 mm visual analogue scale (VAS) where 0 represented no pain and 100 represented unbearable pain, and on a 5-item verbal rating scale with the following options: no, mild, moderate, severe, and very severe pain. Individuals with no or mild pain were classified as nonresponders, and those with moderate or severe pain as pain responders. Patients with very severe pain were advised to withdraw from the study. Pain intensity (VAS) was registered preoperatively and at 20, 40, 60, 80, 100, 120, 140, 180, and 240 minutes after surgery.

### 2.3. Anesthetic procedure

When needed, premedication with midazolam 1–2 mg i.v. was administered 1 hour before induction. General anesthesia was performed using propofol (target-controlled infusion; target, 2–4 μg/mL), and remifentanil (0.1–0.3 μg·kg^−1^·min^−1^ i.v.). Airways were secured with a laryngeal mask and subsequently ventilated with 30%–50% oxygen. Vital parameters were continuously monitored. Fentanyl 1 μg/kg i.v. was administered to prevent remifentanil-induced postoperative hyperalgesia. No other analgesics were used, thus, eliminating confounding factors related to drug-induced pain relief. During surgery, a 20-G intra-articular catheter was placed for synovial fluid sampling and drug administration. The method has been previously described in detail.^28^

### 2.4. Postoperative observations

Over 120 minutes after surgery, all patients were asked to estimate their pain intensity on the 0–100 mm VAS, and on the 5-item verbal rating scale. Individuals who reported no or mild pain until 120 minutes after the completion of surgery were classified as nonresponders. Patients reporting moderate or severe pain were classified as pain responders. Patients with very severe pain were advised to withdraw from the study.

### 2.5. Biobank

Patients who reported pain of moderate or severe intensity at between 0 and 120 minutes after the completion of surgery were included in the biomarker analysis. Synovial fluid samples from the knee joint were drawn through the intra-articular catheter. These synovial fluid samples were assayed for concentrations of relevant inflammatory biomarkers. The results were analyzed for statistically significant sex differences. Individuals with no or mild pain (nonresponders) were not included in the biomarker analysis.

### 2.6. The multiplex proximity extension assay

Synovial fluid was analyzed using the proximity extension assay, which allows simultaneous measurement of 92 biomarkers (Table 1).^16^ The samples were assessed using the Proseek Multiplex Inflammation I panel (Olink Bioscience, Uppsala, Sweden), following the manufacturer's instructions. A 1-μL serum sample was mixed with 3-μL incubation mix (containing 2 antibodies labeled with single-strand DNA oligonucleotides), and incubated at 8°C overnight. We then added 96-μL extension solution (containing proximity extension assay enzyme and PCR reagents), and this mixture was incubated at room temperature for 5 minutes, to allow the formation of DNA-reporter sequences through proximity ligation of the A and B oligonucleotides. Next, the samples were transferred to a thermal cycler for 17 cycles of DNA amplification by quantitative PCR. We then prepared and primed a 96.96 Dynamic Array IFC (Fluidigm, San Francisco, CA) following the manufacturer's instructions. The unique primer pairs for each cytokine were loaded into the left side of the 96.96 Dynamic Array IFC, and the protein expression program was run in a Fluidigm Biomark reader following the instructions for Proseek.

**Table 1:**
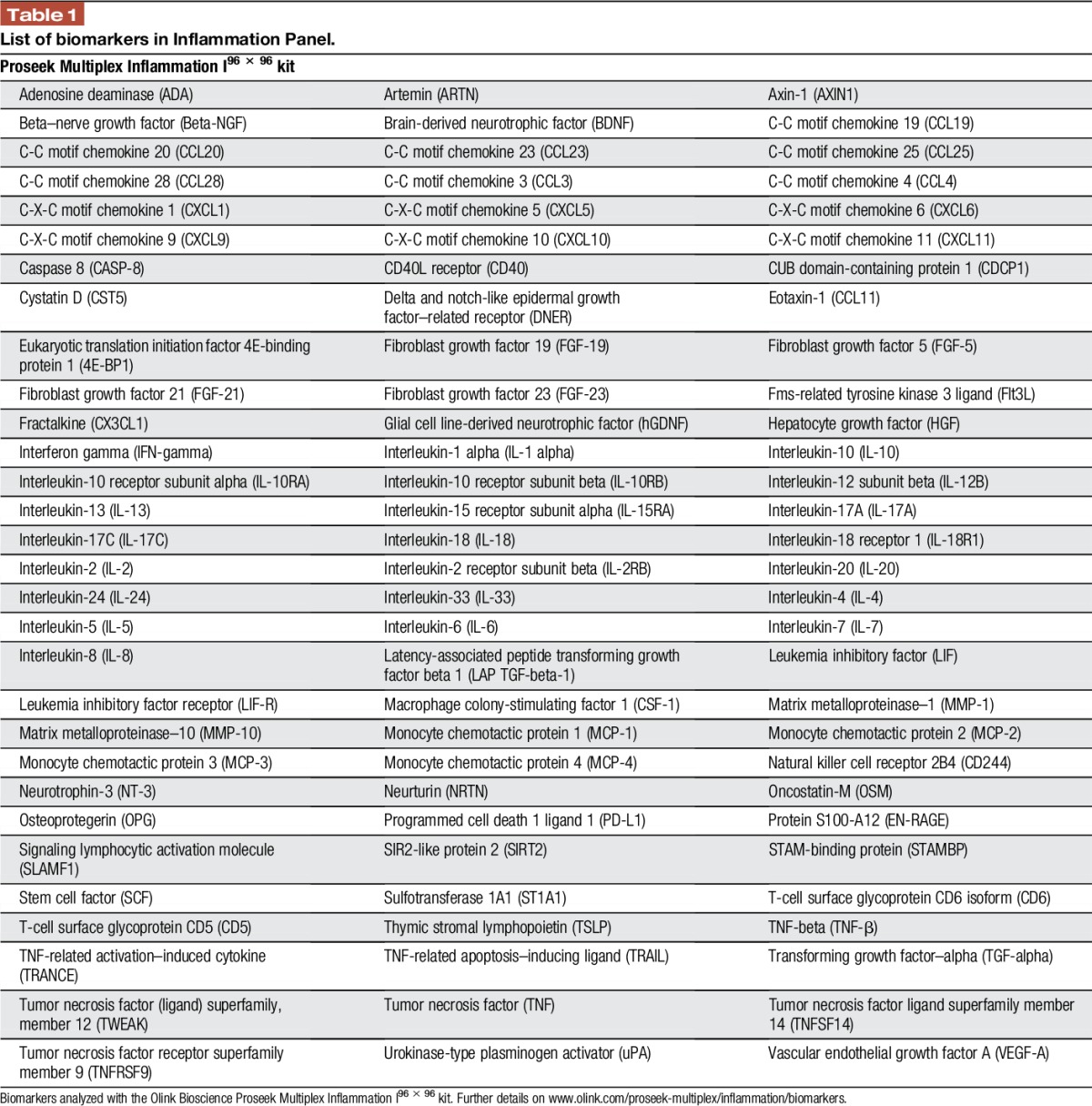
List of biomarkers in Inflammation Panel.

### 2.7. Statistical analyses

Normality was tested using the Kolmogorov–Smirnov test. Baseline characteristics and demographic data are presented as mean (SD) if normally distributed, or as median (range) if not. A double-sided Student *t* test was used to compare mean values for normally distributed data. For nonnormally distributed data, mean values were compared using a nonparametric 2-sided Mann–Whitney *U* test. Pain response was analyzed using a logistic regression model including sex, age, preoperative pain, and surgery duration as possible predictors. Data for each biomarker were evaluated using the statistical software R^36^ and of SPSS version 23 (Statistical Packages for the Social Sciences, Chicago, IL). Protein expression levels were below the laboratory's limit of detection (LoD) for some patients. Hence, to calculate the between-group test differences, we used a multiple linear regression model with inputted LoD values in place of the below-LoD values. Because of the difficulties associated with analyzing data including a high proportion of below-LoD values, biomarkers for which over 25% of the values were below the LoD were excluded from statistical analyses.

The simultaneous analysis of 92 biomarkers increases the risk of false discoveries of significance. Therefore, we made adjustments for the false discovery rate (FDR) using the procedure of Benjamini and Hochberg,^4^ and estimated the difference in expression levels by computing the ratio of the group medians on the original non–log scale. To visualize the group differences, we performed linear discriminant analysis,^10^ based on a weighted average of the differentially expressed proteins. In biomarker measurements, a positive score indicated a high inflammatory activity, whereas a negative score indicated a low inflammatory activity.

## 3. Results

### 3.1. Incidence

Table 2 presents the demographic data, preoperative pain, and distribution of surgical procedures. Among the 65 included patients, 44 (68%; 23 women, 21 men) developed moderate or severe pain. In agreement with previous data, the distribution between pain responders and pain nonresponders indicated that around 30% of the population recovered without requiring analgesics, whereas 82% of women and 57% of men reported moderate or severe postoperative pain requiring active treatment.

**Table 2:**
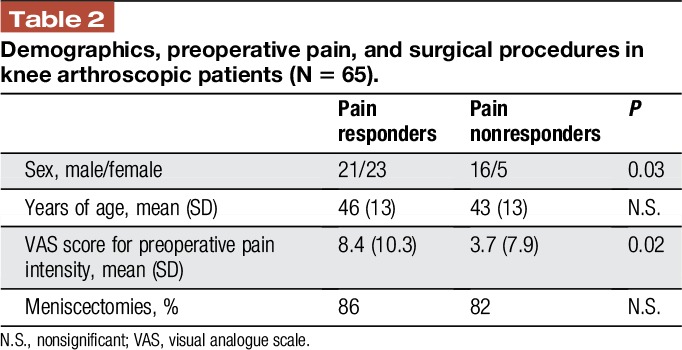
Demographics, preoperative pain, and surgical procedures in knee arthroscopic patients (N = 65).

**Figure 2.**
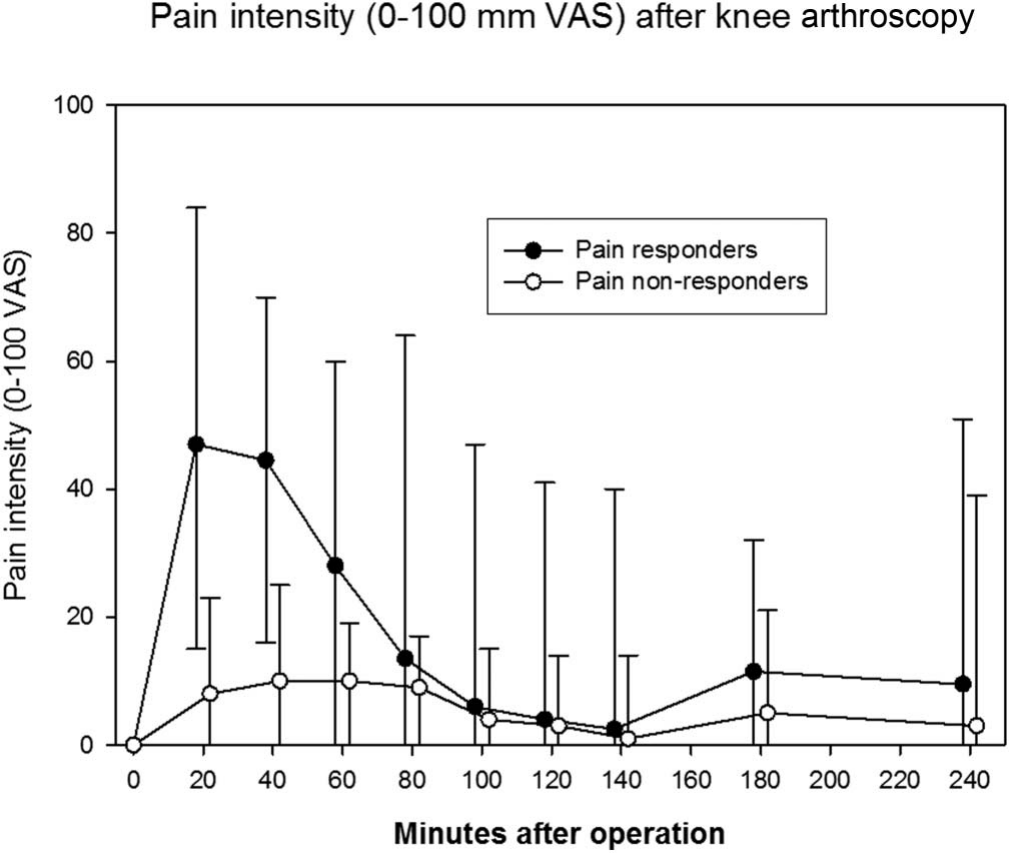
Pain intensity after knee arthroscopy based on a visual analogue scale (VAS) ranging from 0 (no pain) to 100 mm (unbearable pain).

Logistic regression analysis was performed to examine the effects of sex, age, preoperative pain, and surgery duration on the likelihood of patients developing moderate or severe pain. Women were 4.9 times more likely to report moderate or severe pain than men (95% confidence interval [CI], 1.2–19.6, *P* = 0.024). Patient age, preoperative pain, and surgery duration were not statistically significant factors. Among pain responders, the mean VAS score was 46.4 mm among women (95% CI, 39.4–53.3 mm) and 39.7 mm (95% CI, 30.9–48.4 mm) among men, and mean time until inclusion was 23 minutes for women (95% CI, 14–31 minutes) and 21 minutes for men (95% CI, 16–26 minutes) (nonsignificant difference). One patient reported very severe pain, and was offered exclusion but decided to continue participation. The pain responders experienced a peak pain intensity, followed by a regression to pain intensity values comparable with those reported by nonresponders (Fig. 2).

### 3.2. Multiplex cytokine analysis

Biomarker levels were measured in synovial fluid samples from 58 patients. The samples from 44 patients yielded at least 75% of the desired measurements (Fig. 1). These data were statistically compiled and evaluated using the 5% FDR method and linear discriminant analysis.

**Figure 3.**
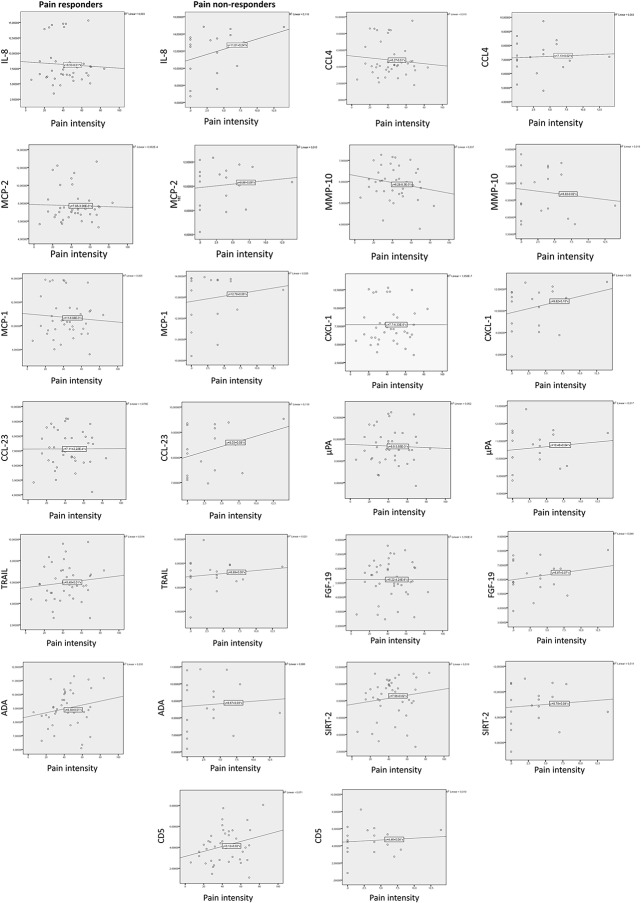
Scatterplots with linear regression line of pain intensity (VAS) and the 13 biomarkers with NPX difference >0.5 in pain responders and pain nonresponders. ADA, adenosine deaminase; NPX, normalized protein expression; TNF, tumor necrosis factor; TRAIL, TNF-related apoptosis–inducing ligand; VAS, visual analogue scale.

Among the pain responders, normal distribution could be assumed for all proteins; therefore, a Welch test was used for analysis. After correction for a 5% FDR, the only significant sex-based difference was that MMP-10 levels were higher among men (*P* = 0.01). Linear discriminant analysis revealed that 3 proteins (IL-8, CCL-4, and MCP-2) were higher in men, with differences of >1 normalized protein expression (NPX). No proteins were overexpressed by >1 NPX in women. A difference of at least 0.5 NPX, which can be considered to represent a biological change, was found to indicate male overexpression of all included proteins—except for adenosine deaminase, SIRT-2, and CD-5, which were slightly higher among women (Table 3). However, pain intensity was not significantly correlated with the NPX values for these biomarkers (Fig. 3).

**Table 3:**
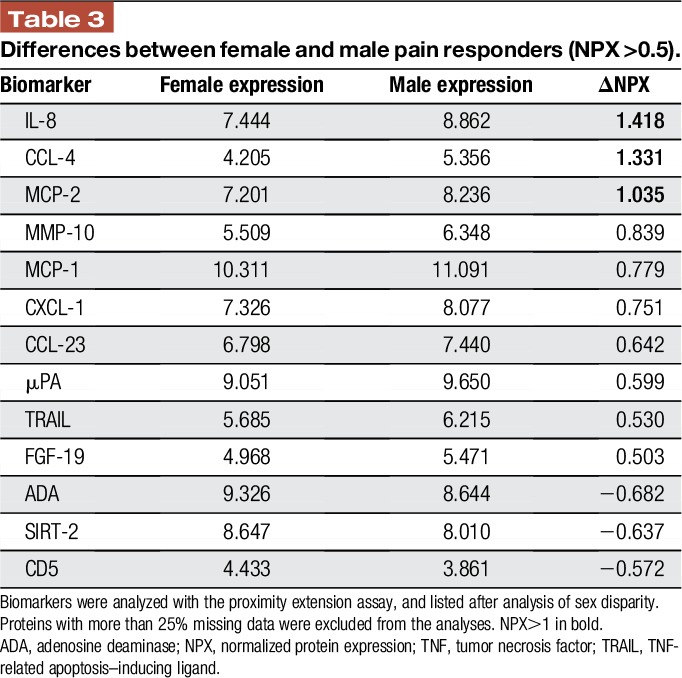
Differences between female and male pain responders (NPX >0.5).

## 4. Discussion

Our present results suggest that postoperative pain was perceived by a significantly higher proportion of women than men (82% vs 57%). Of interest, this difference was not reflected by the biomarker analyses, as men showed higher levels of proinflammatory cytokines. Our analysis of 92 biomarkers did not enable identification of peripheral inflammatory mechanisms that contribute to the generally greater prevalence of pain in women compared with men.

### 4.1. Prevalence and sex inequalities of pain after surgery

Previous studies have consistently documented a significant divergence between male and female pain responses.^7,26^ Moreover, experimental pain studies show apparent differences in how men and women report pain. Studies using the cold pressor test report lower tolerance and endurance among women. Although female subjects show lower tolerance for heat and pressure stimuli, ischemic pain tolerance is comparable between the sexes.^24^ Our present findings that moderate or severe pain after knee arthroscopy was more frequent among women are comparable with previous data.^25,27,28,32^

Sex-related pain variability may be partly due to the influence of sex hormones.^9^ Numerous studies have examined pain in relation to the menstrual cycle.^6,31^ One limitation of this analysis is that we did not record menstrual cycle phase. However, in a comprehensive review, Klatzkin et al.^12^ reported that menstrual cycle phase plays a negligible role in explaining fluctuations in pain, with the possible exception of experimental electrical pain stimulation.

Pain intensity after knee surgery may be influenced by preoperative pain intensity.^14^ The patient population in our study was generally healthy and reported low preoperative pain intensity. Pain responders experienced slightly higher preoperative pain intensity than nonresponders. This difference was statistically significant but of limited clinical significance. Preoperative pain was excluded from the final logistic regression model of pain responsiveness because of a lack of statistical significance.

It has been suggested that psychosocial factors may contribute to the pain experience, with cultural and social sex roles potentially influencing pain sensitivity, endurance, and willingness to report.^2^ This is supported by studies using the cold pressor test for experimental pain induction, with findings consistent with stereotypical preconceptions that women present lower pain tolerance and higher vigilance to perceived pain than men.^11,21^ Future studies should consider the potential bias induced by these confounding factors regarding reported pain in clinical trials.

### 4.2. Biochemical signaling in acute inflammation

Cytokine signaling comprises a vast number of cascades that stimulate or inhibit the recruitment of humoral responses and modulate nerve transmission. In this trial, we measured important peptides that increase during the early postoperative phase, in relation to inflammation and pain. Our analysis of 92 cytokines of presumed importance represents a valuable exploratory investigation. However, this study did not include all potentially interesting biomarkers, thus leaving potential mediators undiscovered. The hypothesis was that acute pain, which occurs to a greater extent in women than men, would be reflected by diversity in inflammatory biomarkers. However, these results did not support this theory. In contrast, men showed higher levels of proinflammatory cytokines IL-8, CCL-4, and MCP-2. Few studies have examined human cytokines and chemokines and their contributions to pain physiology after joint injury.^20^ The unexpected findings in this study contribute important information to this field of research.

In addition to the proinflammatory cytokines/chemokines, men also showed higher levels of MMP-10. Matrix metalloproteinases are involved in cartilage remodeling in pathologic joint conditions, such as posttraumatic osteoarthritis, and are capable of degrading virtually all extracellular matrix components.^13^ They can also potentially stimulate or inhibit the cytokine-derived inflammatory response through cleavage of proinflammatory mediators (eg, IL-1β, IL-8, and TNF-α) and can reportedly induce downstream events, such as leukotaxis and hyperalgesia.^1,17,23^ MMP-10 shows catalytic activity on various collagens and fibronectin, and is involved in cellular migration and resorption in developing bone.^13^ In a comparable patient population, total MMP activity was elevated nearly 25-fold in the synovial fluid of knees with meniscus tears compared with in control knees,^15^ although MMP-10 level was not increased. Experimental data in macrophages indicate that MMP-10 moderates the response to acute infection by inhibiting the proinflammatory response.^19^ Here, we analyzed MMP-1 and MMP-10 (Table 1) and found that only MMP-10 levels significantly differed between sexes. In this study, it is impossible to elucidate whether MMP-10 exerts activating or inhibitory effects on pain because the sampling time disparity prohibited comparison between the pain responders and the pain nonresponders. This limitation could theoretically be overcome by designing a trial that involved synovial fluid sampling before surgery. Analysis of total MMP activity and gene expression levels both before and after surgery would enable better characterization of the possible association between pain and MMP activity.

### 4.3. Limitations

This analysis of data from a small acute pain trial can be useful for hypothesis generation, but the external validity is limited. Day-case surgical patients remain in the hospital for only a few hours, allowing for a limited number of additional tests and questionnaires for research purposes. Only a small number of synovial fluid samples were analyzed. Moreover, the 92 evaluated biomarkers do not constitute a comprehensive analysis of the peripheral mechanisms underlying biochemical expression. Additional postoperative observational studies are needed before dismissing the possibility of sex-specific cytokine signaling, as disparities in inflammatory mediator expressions may still exist.

### 4.4. Conclusions

Acute pain after knee arthroscopy was more intense in women compared with men. In contrast, men showed higher levels of proinflammatory biomarkers and MMP-10. Greater knowledge of cytokine function is needed before concluding that the disparities in biomarker expression are clinically unimportant. Based on the similar biochemical signaling in women and men, it seems that central mechanisms are of greater importance in sex-specific joint pain perception.

## Disclosures

The authors have no conflicts of interest to declare.

This study was financially supported by the Regional Health Authorities of South East Norway, and by Uppsala Berzelii Technology Centre for Neurodiagnostics.
